# Delayed brachial artery reconstruction after traumatic injury: a case for sustainment of surgical intervention

**DOI:** 10.11604/pamj.2017.27.232.7291

**Published:** 2017-07-31

**Authors:** Kelechi Emmanuel Okonta, Emmanuel Ossai Ocheli, Tombari Joseph Gbeneol

**Affiliations:** 1 Department of Surgery, University of Port Harcourt Teaching Hospital, Rivers State, Nigeria; 2 Federal Medical Centre Owerri, Nigeria

**Keywords:** Brachial artery, delayed, injuries, reconstruction

## Abstract

The brachial artery is the commonest artery injured in the extremities. Although the patients present late, nevertheless reconstructions is advocated in other to salvage the limb and maintain function of the hand. We retrospectively examined 25 consecutive patients with vascular injuries treated at The Cardiovascular and Thoracic Surgery Unit of a tertiary health centre over a period of 4 years. We assessed the pre-tertiary methods of stopping of bleeding injured brachial arteries, mechanisms of injury, associated injuries, treatment and the outcome following vascular repair in terms of functionality of the forearm and the volume of the radial pulsation. A total of 12 patients (48.0%) had brachial artery injuries out of the 25 patients with different forms of vascular injuries during the period. There were 10 males and 2 females, aged 7.5-65 years. The aetiology of the brachial artery injuries were: Glass cut in 5 patients, knife cut in 3 patients, surgical complication of tendon release (iatrogenic) in 1 patient, injury from self injection of pentazocine in 1 patient, machete cut in 1 patient and blunt vascular injury from fan belt injury in 1 patient. Except for the young girl whose brachial artery was injured at surgery, and had lateral repair done within 3hours, the timing between injury and repair in the remaining 11 patients ranged between 6-288 hours. This was beyond the golden time in trauma cases. Two patients had the brachial artery revascularised using the Reversed Saphenous Vein Graft (RSVG). The wrist pulsation was small volume in one patient as felt by palpation before discharge though the forearm was viable. Otherwise the remaining patients’ outcome was good. Most of the patients with brachial artery injury present late following injury. Revascularisation beyond the golden hour is still desirable as it will help to prevent limb loss. Plans should be put in place to train vascular surgeon to encourage prompt and expertise care.

## Introduction

Vascular injuries in the country pose a great deal of distress to patients as a result of dearth of vascular surgeons to deal with the challenge [1]. This is more often than not, leads to delayed presentations for specialist care [2, 3] with a poor outcome. It has been observed that patients with trauma have better outcomes if they are given definitive care within 1 hour of the occurrence of their injuries generally [4]. For vessels, there is the need to revascularised the injured vessel to improve outcome in terms of function and salvaging of the limb. The brachial artery is the most frequently injured artery in the upper limb [5, 6]. Yet most of the patients present late [2, 3, 6] when surgical intervention would have been thought not to be necessary. The repair of traumatic brachial artery injury beyond the ‘golden hour’ is still recommended [7]. It is advocated that prompt repair be effected as this is necessary for the survival of the patient and salvage of the limb [5]. Patients with traumatic brachial artery injury have delayed referral to the tertiary centres for arterial reconstruction in our practice. We review the early outcome of delayed surgical reconstruction of brachial artery injuries in these patients. The essence of vascular surgery is to save limb and preserve function.

## Methods

We retrospectively examined 25 consecutive patients with vascular injuries of the extremities treated at the Cardiovascular and Thoracic Surgery Unit of two tertiary health centres where the consultants worked over a period of 4 years. We assessed the mechanisms of injury, associated injuries, treatment and the outcome following vascular repair in terms of functionality of the forearm and the volume of the radial pulsation.

### Pre-tertiary hospital center methods of stopping bleeding

The options used in stopping bleeding at peripheral hospitals or referral health centers prior to presentation at the tertiary hospital were the application of tourniquet, use of firm dressing over the lacerated area, use of forceps to hold the bleeding vessels and ligation of the bleeding vessel.

### Surgical technique

**Figure 1:**
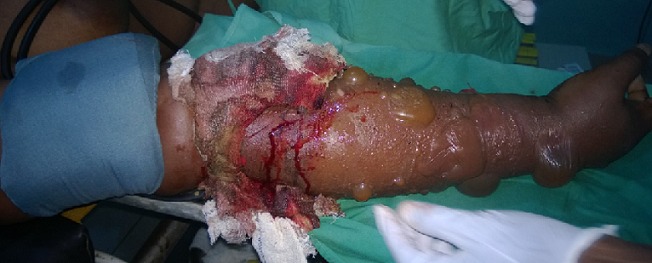
Injured forearm

**Figure 2:**
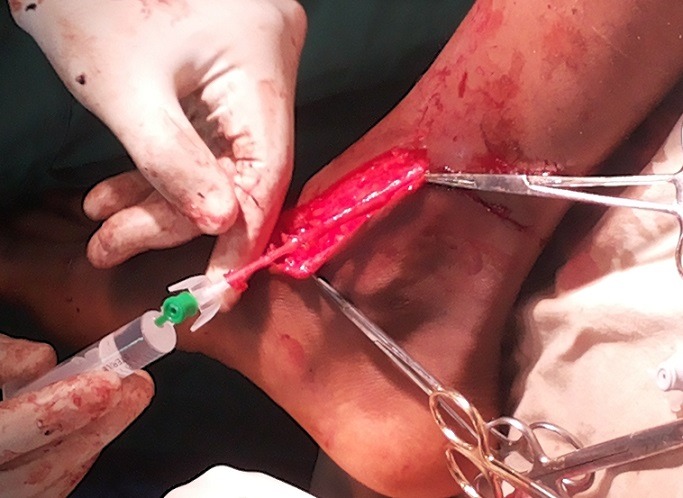
Greater saphaneous vein harvest

**Figure 3:**
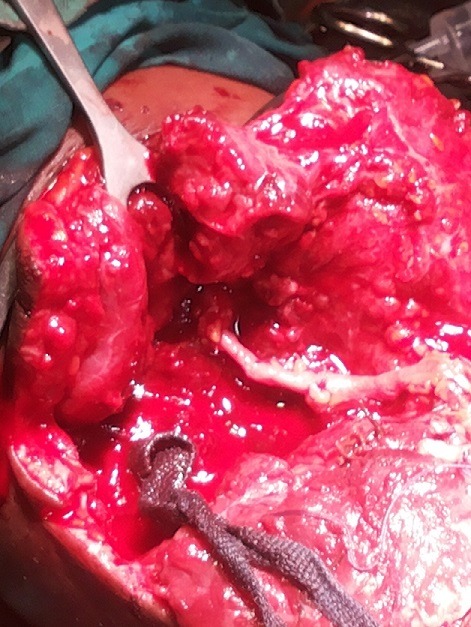
Proximal anastomosis

**Figure 4:**
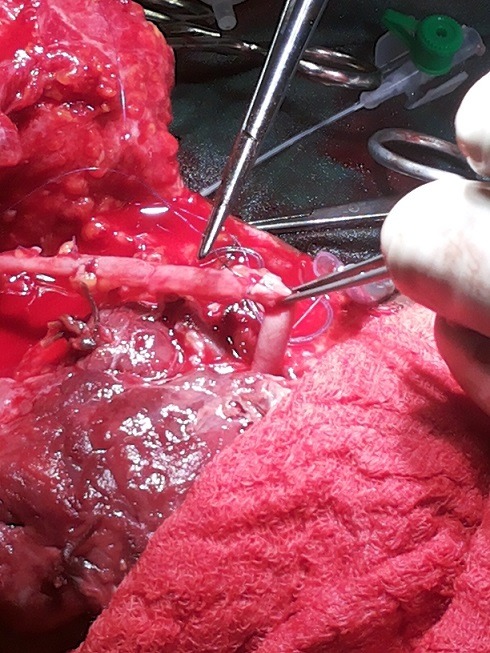
Distal anastomosis

The diagnoses were made by mainly clinical evaluations while in some cases Doppler ultrasonography was used especially for the blunt vascular injuries and during post operative periods. The operations were done under general anaesthesia and the mercury sphygmomanometer was employed in place of Esmarch’s tourniquet to control active bleeding and provide intermittent, regularised limb tourniquet. Distal embolectomy was done with either fogarty catheter or improvised small – sized (size 6Fr) Foley urethral catheter followed by intravascular irrigation using heparinised solution (5,000in in 50mls) [8] through a canula of fair size. For patient requiring vein graft the leg was prepared and the great saphenous vein harvested and put in gallipot containing heparinised solution and papaveratum or lidocaine. Prolene 6/0 was used for the repairs (Figure 1, Figure 2, Figure 3, Figure 4). The success of revascularization was immediately assessed by feel for the return of the radial pulsation. Therapeutic antibiotics, analgesic, aspirin tablets and subcutaneous heparin were continued post - operatively in the patients.

## Results

A total of 12 patients (48.0%) had brachial artery injuries out of the 25 patients with different forms of vascular injuries during the period (Table 1). There were 10 males and 2 females, aged 7.5-65 years. The aetiology of the brachial artery injuries were: Glass cut in 5 patients, knife cut in 3 patients, as a surgical complication during tendon release (iatrogenic) in 1 patient, injury from self injection of pentazocine in one patient, machete cut in 1 patient and blunt vascular injury from fan belt injury in 1 patient. (Diagram 1-4). It was only the young girl whom the brachial artery was injured at surgery who had lateral repair done within 3 hours while the time interval between brachial artery injury and repair in the remaining 11 patients ranged between 6-288 hours. Six patients had end to end anastomosis,3 patients had lateral repairs and 2 patients had the brachial artery revascularised using the RSVG. The use of forceps or ligature to stop bleeding before patients reported to the hospital did not affect the modality of repair in terms of the use of RSVG or end-to-end anastomisis. The wrist pulsation was small volume in one patient as felt by palpation before discharge though the forearm was viable. Otherwise the remaining patients’ outcome was good.

**Table 1:** Patients with brachial artery injuries out with different forms of vascular injuries during the period

S. No	Initial	Age	Sex	Aetiology	Artery	Timing	Treatment	Outcome
1	PO	33	F	Selfinjection	RBA	240hrs	Lat Repair	Good
2	EJ	27	M	Glass cut	LBA	6 hrs	RSVG	Good
3	FJ	7.5	F	Iatrogenic	LBA	3hrs	Lat Repair	Good
4	UG	25	M	Knife Cut	LBA	288hrs	RSVG	Good
5	UM	65	M	Knife Cut	LBA	21hrs	E-n-E	Good
6	EO	47	M	Blunttrauma	RBA	11hrs	E-n-E	Good
7	AJ	25	M	Knife Cut	RBA	11hrs	Lat Repair	RP-not
8	GJ	60	M	Glass Cut	RBA	8hrs	E-n-E	Good
9	ND	35	M	Glass Cut	RBA	7hrs	E-n-E	Good
10	OL	8	M	Glass cut (Beer bottle)	LBA	7 hrs	E-n-E	Good
11	I P	35	M	Glass Cut (Beer bottle)	LBA	20hrs	E-n-E	Good
12	UK	29	M	Machete	RBA	48	E-n-E	Good

## Discussion

Most of the vascular injuries were caused by penetrating injuries from glass and knife cuts. One patient had blunt trauma when a fan belt from a grinding machine from a grinding machine hit his forearm. He sustained endothelial injury with associated intra-luminal thrombose formation in the brachial artery for which arteriotomy, embolectomy and lateral repair were done. Stab wounds and blunt trauma were the commonest modes of injury as observed in a study in our country about 3 decades ago [2]. In another country outside, stab injuries were the commonest while other causes included glass cut injuries, industrial accidents, road traffic crash, gunshots [9, 10]. One of the patients with penetrating vascular injury was from self injection of pentazocine which led to vascular laceration. The consequences of recreational drug use resulting in vascular injury and posing unique and challenging problems has been previously reported [11]. When vein graft was required, we preferred the saphenous graft as against the cephalic graft suggested by some authors [12] which in our view will affect the drainage of the upper limb especially the affected limb and technically more difficult than the greater saphenous vein to harvest.

In our study, most of the patients with traumatic brachial artery injuries had delayed presentation in our setting, thus making delayed arterial revascularization the treatment norm. This was the finding in other centers [2, 5]. So, it stands to reason that repair can be effected even beyond the golden hour [1]. This is unarguably so because no critical limb ischaemia occurred in any of the patients even after delayed revascularisations. This was the submission of Zellweger et al on the analysis of 124 patients with brachial artery injury whereby they stated that critical limb ischaemia rarely occurred [9]. The data available recommend brachial arterial repair in patients with traumatic artery injury even after golden time of arterial repair [7]. Upper limbs were saved in the remaining 26 cases (96%) [7]. Critical limb ischemia rarely occurred [9]. Equally Simmon et al specifically stated that delayed presentation greater than 6 hours amongst other things were not predictive of amputation for patients with brachial artery injuries [13]. Though prompt repair of traumatic brachial artery injuries is important to prevent compartment syndrome , which can lead to functional deficits [5]. However, when there is need, prophylactic fasciotomy should be considered [6]. The implication of this is that there is no need to waste time when the patients present for brachial artery revascularisation

One of the surgical options is reverse autogenous interpositions grafts using the saphenous or cephalic vein [12] when the defect is wide and requires bridging it up. Synthetic graft can also be used but the added cost in procuring the material in out setting is a discouraging factor. The other option is end to end anastomosis in a defect that is not wide, in which the anastomosis must not be under tension after repair. There is the lateral repair when there is lateral laceration or following arteriotomy to evacuate intraluminal clot as we did in one of the patients with blunt vascular injury in which intraluminal thrombose was formed.

The use of forceps or ligature to stop bleeding before patients reported to the hospital did not affect the modality of vascular repair in terms of the use of RSVG or end-to-end anastomisis. That manoeuvre actually prevented the exsanguinations of the patient. However, in other to achieve tension free anastomosis after cutting off the ligature or re-freshened ends of the vessel, both distal and proximal anastomosis were done and back slab put in some cases with the forearm slightly flexed.

## Conclusion

Most of the patients with brachial artery injury present late following injury. Revascularization beyond the golden hour is still desirable to prevent limb loss and restore function. Plans should be put in place to train vascular surgeons to encourage prompt and expertise care.

### What is known about this topic

- Brachial artery injury is the commonest artery affected in the limbs;
- Brachial artery injuries lead to upper limb loss;
- Patients with brachial artery injuries present late.

### What this study adds

- Brachial artery injury can be repair beyond the golden rule time;
- Brachial artery injury repair has good outcome;
- Delayed brachial artery repair prevents limb amputations.

## Competing interests

The authors declare no competing interest.
